# Therapeutic efficacy of trehalose eye drops for treatment of murine dry eye induced by an intelligently controlled environmental system

**Published:** 2012-02-04

**Authors:** Jinyang Li, Christophe Roubeix, Yu Wang, Shuai Shi, Guoting Liu, Christophe Baudouin, Wei Chen

**Affiliations:** 1 School of Ophthalmology and Optometry, Wenzhou Medical College, Wenzhou, Zhejiang, China; 2 INSERM U968, Université Pierre et Marie Curie Paris 6, Institut de la Vision, Paris, France; 3 Department of Ophthalmology, Quinze-Vingts National Ophthalmology Hospital, Paris, France; 4 Ambroise Paré Hospital, AP-HP, University of Versailles Saint-Quentin en Yvelines, Versailles, France

## Abstract

**Purpose:**

To determine whether eye drop instillation of the disaccharide trehalose (TT) alleviates ocular surface damage in a dry eye murine model.

**Methods:**

Dry eye was induced in mice using an intelligently controlled environmental system (ICES). After 21 days housed in the ICES without topical treatment, the mice were randomly divided into three groups: no eye drops (ICES) for three weeks, four times a day with PBS 0.01 M 10 µl/eye bilaterally (ICES+PBS), or with TT 87.6 mM 10 µl/eye bilaterally (ICES+TT). Another mice group that was not exposed to the ICES and received no treatment served as a control group (UT). The ocular surface integrity, in each group, was evaluated using Oregon Green dextran (OGD) and fluorescein staining. The expression and distribution of occludin, involucrin, and small proline-rich protein 2 were determined with immunohistology analysis on whole mounted corneas. Heat shock protein 70 (HSP70) and matrix metalloproteinase 9 (MMP-9) expression was estimated with immunohistology. Ocular surface inflammation associated with each treatment was estimated with real time-PCR of interleukin-1β (IL-1β), IL-2, IL-6, IL-17, and tumor necrosis factor-alpha in the conjunctiva.

**Results:**

OGD staining in the cornea epithelium was lower in the ICES+TT group than in the ICES and ICES+PBS groups. Corneal epithelial occludin staining was markedly more homogenous in the ICES+TT group than in ICES and ICES+PBS groups, and there were no desquamating apical epithelial cells. Involucrin and small proline-rich protein 2 labeling of whole mounted corneas revealed upregulation of their expression in the groups, which received no treatment or PBS instillation compared to the ICES+TT group. HSP70 and MMP-9 immunolabeling revealed a marked increase in corneal epithelial expression in response to the ICES. The group treated with trehalose showed a similar profile expression of HSP70 and MMP-9 as the control group (UT). Conjunctival *IL-1β*, *IL-2*, *IL-6*, *IL-17*, tumor necrosis factor-alpha (*TNF-α*), and *MMP-9* mRNA expression was lower in the ICES+TT group than in the ICES or ICES+PBS group.

**Conclusions:**

Trehalose application restored ocular surface integrity, suppressed inflammatory and proteolytic MMP-9 and HSP70 expression, and keratinization in mice with dry eye damaged by a desiccative model.

## Introduction

Over the past 10 years, substantial progress has been made in identifying dry eye disease (DED) pathophysiology. DED is now recognized as a multifactorial disease resulting in inadequate hydration of the ocular surface leading to tear film hyperosmolarity and eventually to clinical symptoms of discomfort, declines in ocular surface epithelial renewal, and visual disturbance [1]. Hyperosmolarity is known to induce ocular surface inflammatory responses [2], mainly through the activation of receptors that also induce pain. One is the transient receptor potential vanilloid type 1 (TRPV1) whose activation by hypertonicity in cell culture induces inflammatory responses [3]. These biologic consequences account for the ocular surface injury reported in more than 80% of patients with DED altering their quality of life [1].

Whatever the level of dry eye severity, frequent applications of artificial tears remain a widely accepted therapy for DED. Used over the past 30 years, this treatment has proved that it is able to provide noteworthy symptomatic relief [4]. Nevertheless, the remaining challenge is to improve tear film supplementation efficacy by including more efficient active ingredients or excipients to optimize ocular moisturizing and prevent epithelium injury.

Trehalose (TT) is a non-reducing disaccharide of glucose naturally widespread in many living organisms, including plants, insects, fungi, and bacteria. Trehalose has been identified as a key response element needed for survival during exposure to stress-induced desiccation. Trehalose decreases damage to cells caused by desiccation by serving as a “water replacement” or rearranging the intracellular water structure [5]. Matsuo et al. [6] first explored the potential beneficial effect of trehalose on human cells in DED stressed by desiccation. Furthermore, Cejková et al. described a protective effect of trehalose on ultraviolet B–induced corneal damage in terms of oxidative stress, apoptosis [7], and inflammation [8]. We recently showed in a murine dry eye model designated as an ICES that trehalose improves ocular surface epithelial health in vivo during exposure to desiccation through declines in apoptosis [9]. However, a more extensive characterization of the effects of exposure to ICES desiccation on ocular surface epithelial disorders has not yet been well investigated.

We describe here in mice exposed to the ICES that application of trehalose containing eyedrops alleviates DED corneal damage to tight junction proteins and decreases epithelial cornification as well as conjunctival inflammation. Taken together, our findings strengthen the notion that trehalose application may provide an improved therapeutic strategy for DED.

## Methods

### Animals

All procedures were approved by the Animal Care and Ethics Committee of Wenzhou Medical College, Zhejiang, China, and adhered to the Association for Research in Vision and Ophthalmology Statement for the Use of Animals in Ophthalmic and Vision Research. A total of 120 female C57BL/6 mice (age range, 4–6 weeks) were supplied by the Animal Breeding Unit of Wenzhou Medical College.

### Murine dry eye model

This study used a novel murine model of dry eye induced by an intelligently controlled environmental system (ICES) [9]. Mice in a control group were maintained in relative humidity, 60%–80%, no airflow, and at 21–23 °C. Dry eye desiccation was created through exposure to relative humidity of 13.1±3.5%, an airflow of 2.2±0.2 m/s, and 22±2 °C.

### Therapeutic regimen

C57BL/6 mice were used in this study. After 21 days housed in the ICES without topical treatment, mice were randomized into three groups: a) a group that did not receive any topical treatment (ICES); b) a group that received 10 µl/eye 0.01 M PBS (0.01 M, pH 7.4; Maixin Technology, Fujian, China) bilaterally four times a day (ICES+PBS); and c) a group that received 10 µl/eye trehalose (30 mg/ml, 87.6 mM; Théa laboratories, Clermont Ferrand, France) bilaterally four times a day (ICES+TT). Both treated groups received topical eye drops on the same daily regimen for 3 weeks. Normal C57BL/6 mice not housed in the ICES that did not receive any topical treatment (UT) were used as controls.

### Corneal fluorescein and Oregon Green dextran staining

Corneal epithelial staining with Oregon Green dextran (OGD; 70,000 MW; Invitrogen Inc., Grand Island, NY) was assessed in the three different groups. Briefly, 0.5 μl of 50 μg/ml OGD was instilled in the ocular surface 1 min before euthanasia. Corneas were rinsed with 2 ml PBS and photographed with a stereoscopic zoom microscope (V20; Zeiss with krypton-argon and He-Ne laser; Carl Zeiss Meditec, Ltd., Thornwood, NY) under ﬂuorescence excitation at 470 nm. Images were obtained 2 h after instillation of the last treatment drop and were processed. The fluorescein solution contained 1 mg fluorescein sodium in 0.5 ml PBS. The severity of corneal OGD staining shown in digital images was graded by two masked observers, using the Baylor grading scheme for corneal ﬂuorescent staining. The number of fluorescein staining dots were graded in the 1-mm central cornea zone of each eye, on a standardized five-point scale (0 dot, grade 0; 1–5 dots, grade 1; 6–15 dots, grade 2; 16–30 dots, grade 3; 30 dots, grade 4). One point was added to the score if there was one area of confluent staining, and two points were added for two or more areas of confluence.

### Immunofluorescent staining

Occludin, involucrin, and small proline-rich protein 2 (SPRR-2) were evaluated with laser scanning confocal microscopy in whole mounted corneas, and heat shock protein 70 (HSP70) and matrix metalloproteinase 9 (MMP-9) were evaluated in tissue sections.

The eyes of mice from each group were excised, embedded in optimal cutting temperature compound (OCT compound; VWR, Suwanee, GA), and flash frozen in liquid nitrogen. Sagittal 8-µm sections were cut with a cryostat (HM 550; Microm, Waldorf, Germany) and placed on glass slides that were stored at –80 °C. The whole corneas from each group (three corneas/group per experiment, in three different sets of experiments) were freshly harvested. Tissues were fixed with either methanol at 4 °C for 10 min (occludin, HSP70, and MMP-9) or acetone at –20 °C for 5 min (involucrin and SPRR-2). After fixation, they were permeabilized with PBS containing 0.1% Triton-X for 10 min. Then they were blocked with 20% normal goat serum in PBS for 45–60 min (HSP70, MMP-9, occludin, and SPRR-2) or 20% normal horse serum (involucrin). Primary polyclonal rabbit antibody against HSP70 (1:100 dilution; Boster Bio-engineering Limited Company, Wuhan, China), polyclonal rabbit antibody against MMP-9 (1:100 dilution; Abcam, Cambridge, MA), polyclonal rabbit antibody against occludin (1:50 dilution, 5 μg/ml; Zymed, San Francisco, CA), polyclonal rabbit serum against SPRR-2 (1:100 dilution of neat serum; Alexis Biochemicals, San Diego, CA), or polyclonal goat anti-involucrin (1:20 dilution, 2 μg/ml; Santa Cruz Biotechnology, Santa Cruz, CA) were applied and incubated for 12 h at 4 °C. Secondary antibodies, Alexa-Fluor 488-conjugated goat antirabbit immunoglobulin G (1:300; Invitrogen, Molecular Probes, Eugene, OR) or Alexa-Fluor 488-conjugated donkey antigoat immunoglobulin G (1:300; Invitrogen Molecular Probes), were then applied and incubated in a dark chamber for 1 h, followed by counter-staining with propidium iodide (1:200 dilution) or 4',6-diamidino-2-phenylindole (1:1,000 dilution) for 30 min.

Whole corneas were flattened on microscope slides and covered with antifade mounting medium (Beyotime Institute of Biotechnology, Shanghai, China), and then coverslips were applied. Cryosections and whole mounted digital images (512×512 pixels) were captured with a laser-scanning confocal microscope (LSM 710; Zeiss with krypton-argon and He-Ne laser; Carl Zeiss Meditec, Sartrouville, France) with 488-excitation and 543-nm emission filters (LP505 and LP560, respectively). They were acquired with a 40/0.75× objective. Images from treatment and control corneas were captured with identical photomultiplier tube gain settings and processed using the microscope software (ZEN 2008; Carl Zeiss Meditec) and image-analysis software (Photoshop CS5; Adobe Inc., San Jose, CA).

### RNA isolation and quantitative real-time polymerase chain reaction

Total RNA from conjunctivas (two eyes/group/experiment) were extracted and pooled from each of the four experimental groups, using the RNA isolation kit according to the manufacturer’s instructions (PicoPure RNA isolation kit, 40 isolations; Arcturus; Applied biosystems, Foster City, CA). The RNA concentration was measured based on its optical density at 260 nm and stored at −80 °C before use. cDNA was synthesized from 1 μg of total RNA using random primer and Moloney Murine Leukemia Virus reverse transcriptase. Quantitative real-time polymerase chain reaction (qRT–PCR) analysis was employed using the SYBR Green PCR Core Reagents System (Applied Biosystems, Paisley, UK) and Applied Biosystems 7500 Real-Time PCR System (Applied Biosystems). The primers are provided in Table 1. Assays were performed in duplicate and repeated three times using different samples from different experiments. The qRT–PCR results were analyzed using the comparative threshold cycle method and normalized with glyceraldehyde 3-phosphate dehydrogenase (*GAPDH*) as an endogenous reference.

**Table 1:** Primer sequences used for qRT–PCR.

**Gene**	**Forward primer**	**Reverse primer**
*IL-1β*	CACAGGAGCAACGACAAAATACCTGTG	TCTTCTTTGGGTATTGCTTGG
*IL-2*	GGACCTCTGCGGCATGTTCT	ACAGTTGCTGACTCATCATCGAATT
*IL-6*	AGATAACAAGAAAGACAAAGCCAGAGTC	GCATTGGAAATTGGGGTAGGAAG
*IL-17*	CTCAACCGTTCCACGTCACCCT	CCAGCTTTCCCTCCGCATT
*MMP-9*	ACGGACCCGAAGCGGACATT	TTGCCCAGCGACCACAACTC
*TNF-α*	TCTACTGAACTTCGGGGTGATCG	ACGTGGGCTACAGGCTTGTCA
*GAPDH*	GCTCTCTGCTCCTCCTGTTC	GACTCCGACCTTCACCTTCC

### Data analysis

The 2-Ct method [10] was used to analyze the relative changes in gene expression from real-time RT–PCR experiments. Statistical comparisons of four groups for real-time PCR were performed with ANOVA (SPSS 13.0, IBM Corporation, NY), with p<0.05 considered statistically significant. The Mann–Whitney test was used to compare the controls (UT versus ICES) and the treatment groups (ICES+PBS versus ICES+TT). p<0.05 was considered statistically significant. Analyses were performed using SPSS 13.0 software.

## Results

### Effects of intelligently controlled environmental system on corneal dye staining

**Figure 1:**
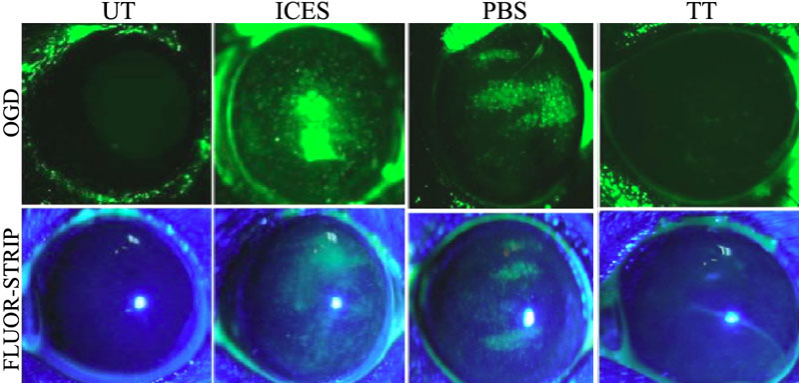
Corneal staining with OGD and fluorescein sodium.

**Figure 2:**
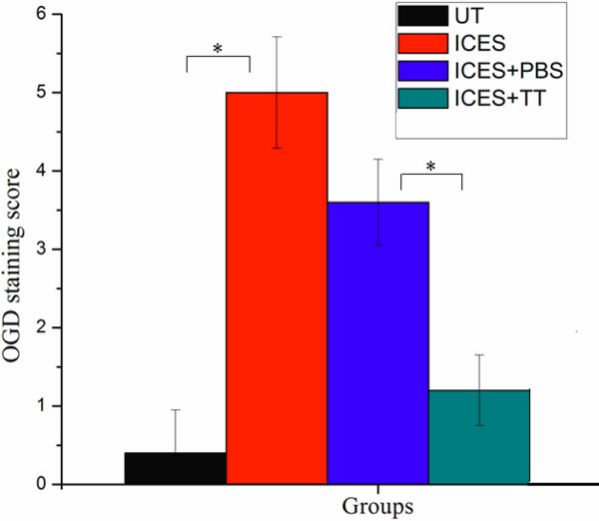
Corneal Oregon Green Dextran staining scores.

To assess changes in corneal epithelial integrity, fluorescein and OGD dye staining were evaluated. Compared with the UT group, corneal uptake of OGD increased in the ICES group. There were more alterations in the cell–cell contacts at the tight junctions and more extensive epithelial desquamation based on more widespread punctuate and confluent staining. On the other hand, the TT group had less corneal staining than either the ICES or the ICES+PBS group. The Baylor grading scheme used to score the staining areas confirmed the protective effect of trehalose (Figure 1, Figure 2, and Table 2). The grading showed the average OGD staining score in eyes treated with trehalose was significantly less than PBS (1.2±0.45 versus 3.6±0.55, p<0.01).

**Table 2:** Oregon green dextran staining scores.

**Group**	**UT**	**ICES**	**ICES + PBS**	**ICES + TT**
OGD staining score (n=5)	0.4±0.55	5.0±0.71	3.6±0.55	1.2±0.45
		p<0.01		p<0.01

Data are shown as mean±SD. Probabilities compare UT versus ICES and ICES + PBS versus ICES +TT.

### Superficial corneal epithelial layer cornification and desquamation

**Figure 3:**
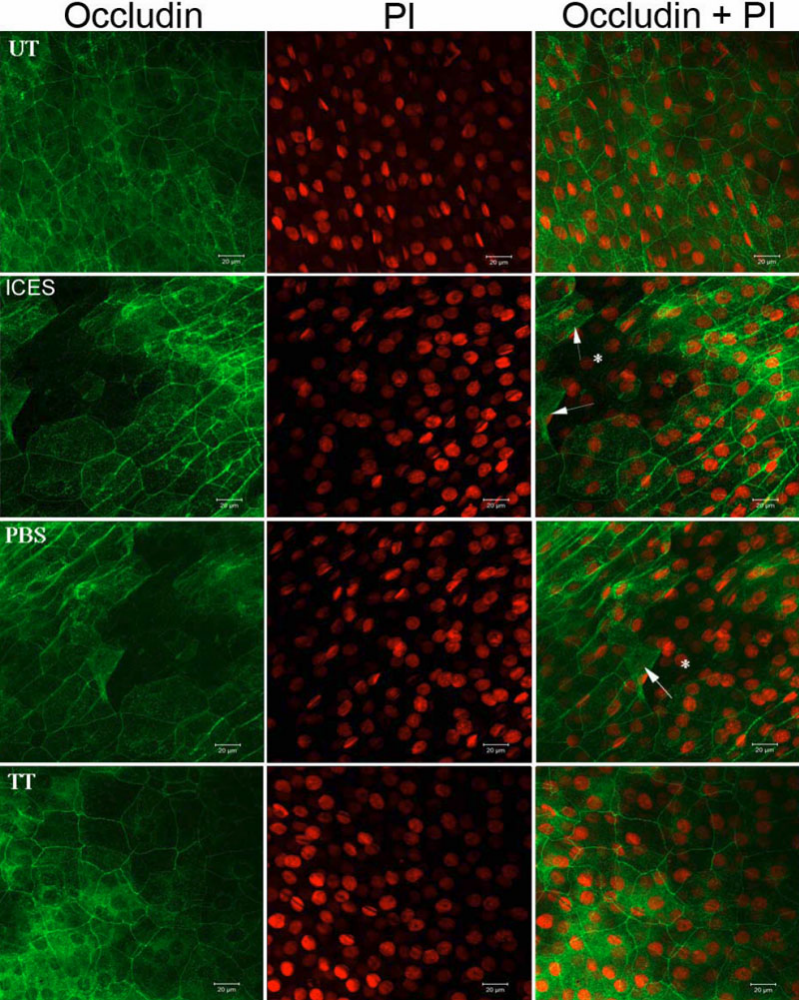
Immunoﬂuorescent staining of whole-mounted corneas stained for occludin. Arrows: desquamating apical epithelial cells; asterisk: holes resulting from the detached cells.

Occludin expression indicates corneal epithelial barrier function and tight junctional integrity. Occludin distribution of whole mounted corneas was presumed to be normal in the UT corneas based on limited fluorescein staining and epithelial cell membrane continuity. Nevertheless, in some peripheral areas, cells were detached from the apical corneal epithelium, but this was rarely seen in the central regions. However, in the ICES group, there was a markedly increased number of desquamating cell areas whereas in the ICES+TT group the corneal epithelium was more homogenous and continuous than in the mice treated instead with PBS (Figure 3).

**Figure 4:**
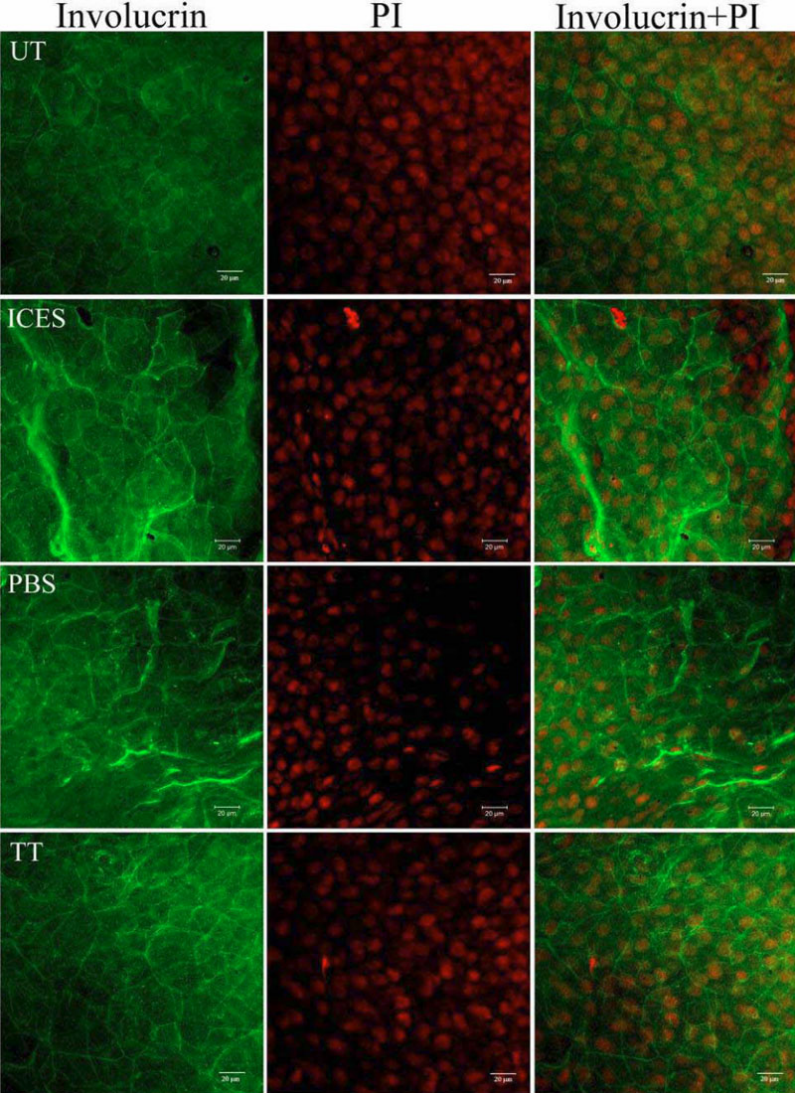
Immunoﬂuorescent staining of whole-mounted corneas stained for involucrin.

**Figure 5:**
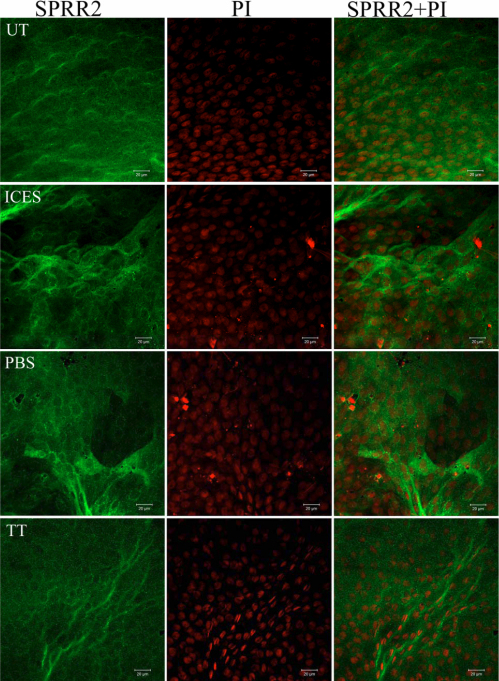
Immunoﬂuorescent staining of whole-mounted corneas stained for SPRR-2.

In whole mount corneas of the UT group, the cytoplasmic confocal images showed that the cornified envelope proteins, involucrin and SPRR-2, were weakly stained. Their labeling in the ICES and ICES+PBS groups was less homogenous with mixed areas of intense and low fluorescence intensity. In contrast, in the ICES+TT and UT groups, both proteins had similar expression profiles (Figure 4 and Figure 5).

### Effects of intelligently controlled environmental system on heat shock protein 70 expression profiles

**Figure 6:**
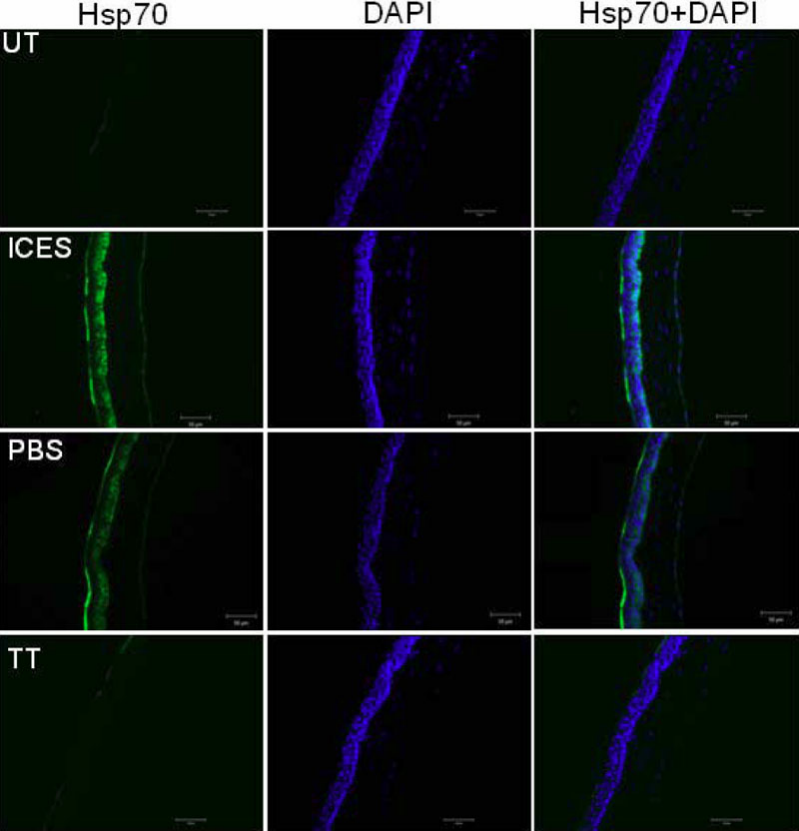
Immunoﬂuorescent staining in tissue sections stained for HSP70.

In the UT group, there was low-intensity HSP70 immunostaining in the apical epithelium. However, in the ICES group HSP70 staining was markedly increased in the apical and basal corneal epithelium. Some of this more intense staining was reduced in the ICES+PBS group. In the ICES+TT group, HSP70 apical epithelium immunostaining was greater than that in the UT group, but lower than in the ICES and ICES+PBS groups. Furthermore, the ICES+TT group had no HSP70 labeling in the basal area of the corneal epithelium (Figure 6).

### Effects of intelligently controlled environmental system on matrix metalloproteinase 9 expression profiles

**Figure 7:**
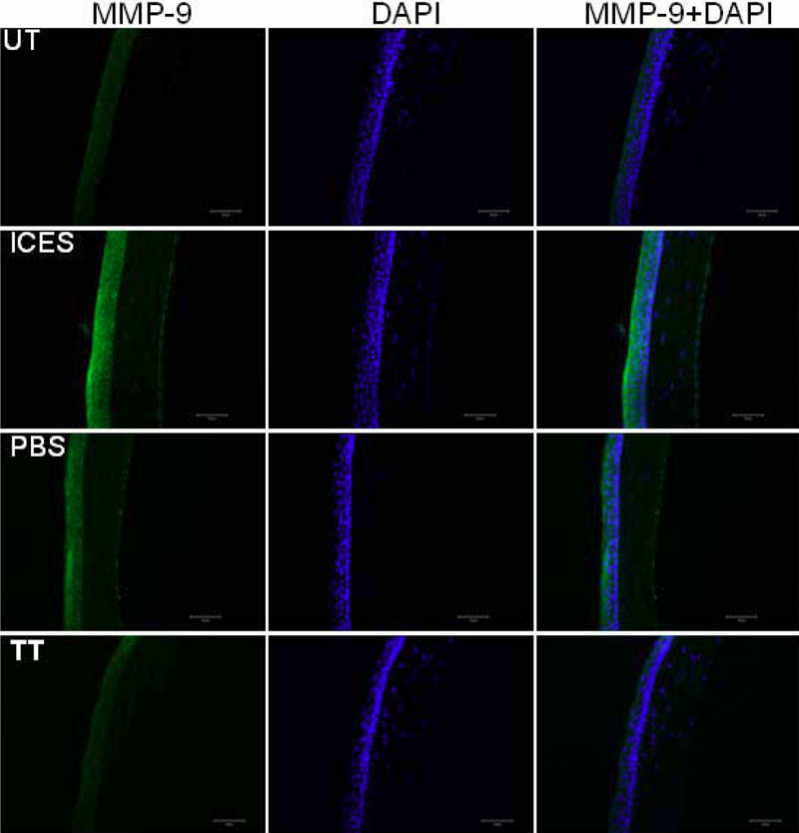
Immunoﬂuorescent staining in tissue sections stained for MMP-9.

MMP-9 immunolabeling in the control UT group was homogenously low in all layers of the corneal epithelium. The staining distribution was the same in the four groups, but with diverse intensities. In the ICES and ICES+PBS groups, staining was consistently more intense than in the UT and ICES+TT groups (Figure 7).

### Effects of intelligently controlled environmental system on inflammatory cytokine and matrix metalloproteinase 9 expression profiles

**Figure 8:**
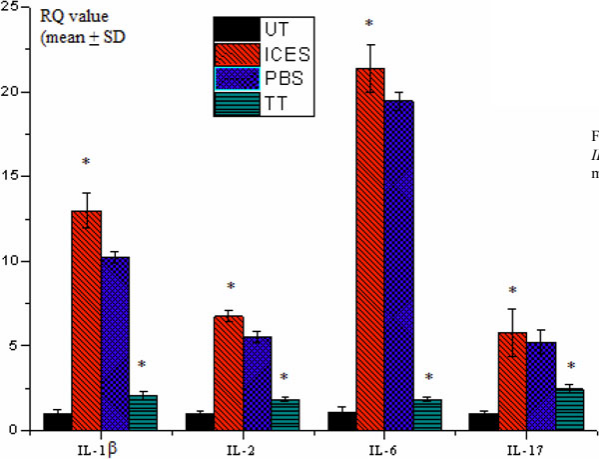
Real-time PCR analysis of *IL-1β*, *IL-2*, *IL-6*, and *IL-17* mRNA in mice conjunctiva.

**Figure 9:**
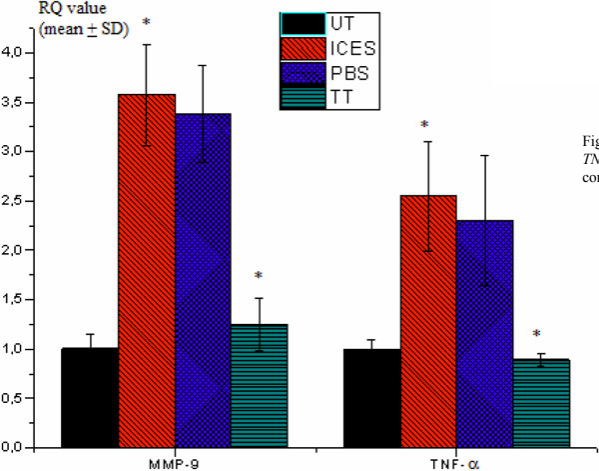
Real-time PCR analysis of *TNF-α* and *MMP-9* mRNA in mice conjunctiva.

Real-time PCR evaluated interleukin-1β (*IL-1β*), *IL-2*, *IL-6*, *IL-17*, tumor necrosis factor-alpha (*TNF-α*), and *MMP-9* mRNA expression levels, which were normalized by the housekeeping gene *GAPDH*. This was done by using pooled total RNA samples of conjunctiva from the UT and the three different ICES groups. The mRNA expression levels for each factor were higher in the conjunctiva of the ICES group than those in the ICES+PBS and the UT as well as the ICES+TT groups. *IL-1β* and *IL-6* mRNA upregulation was particularly pronounced in the ICES and ICES+PBS groups. Trehalose treatment lowered conjunctival MMP-9 expression. Furthermore, the mRNA expression levels of each factor in the ICES+TT and UT groups were not different from one another (Figure 8 and Figure 9).

## Discussion

We showed in a novel murine dry eye model induced with an ICES that eyedrop supplementation with the disaccharide trehalose improves corneal surface hydration and protects against losses in epithelial integrity more than a physiologic solution. Preservation of integrity was attributable to improved organization of epithelial cell junctions, lower proinflammatory cytokine and MMP-9, and gene expression upregulation, presumably due to less pathogenic infiltration, less keratinization, and upregulation of stress markers.

The ICES dry eye model is more representative of environmental challenges to maintaining epithelial integrity since this model mimics the effects of a decrease in ambient humidity that induces dry eye symptomology. Other studies have indicated that the ICES model is relevant because losses in tear film stability described in patients with DED exposed to airflow [11], air pollutants, or dry air at high altitude [12], dry climates mimic those induced in mice by ICES. Dursun et al. [13] showed in a controlled environment chamber model using mice that blockade with scopolamine in a desiccating environment potentiated changes in tear film properties exposed to a continuous airflow. In another study, DED was induced in an environment chamber by controlling temperature and airflow at low humidity without any pharmacological agent injection and/or surgical intervention [14]. The study demonstrated that tear film stability mainly depends on temperature, airflow, and humidity. The ICES model used in this study is an improvement on the controlled environment chamber dry eye model. Our ICES maintains constant airflow, temperature, and humidity by intrinsic cycling, which does not modify the temperature and humidity level. This more efficient desiccant system allowed increased chamber size and animal population [9].

Fluorescein and OGD staining determined ocular surface integrity. The results showed corneal staining was more extensive and pervasive in deeper layers in the ICES than in the UT and ICES+TT groups. The PBS group presented an intermediate score due to the moisturizing property of PBS, which may itself be considered a tear substitute. Less staining in the ICES+TT suggests that this disaccharide reduced losses in epithelial integrity more than that seen in the other groups. Occludin is a major tight junction protein of the corneal epithelium and contributes to support of its barrier function [15,16]. Decreases in occludin staining in the ICES and ICES+PBS groups were most likely responsible for the increase in corneal staining in the ICES group. Depressed barrier function was consistent with more desquamating apical epithelial cells in the ICES and ICES+PBS groups compared to the UT and ICES+TT groups.

Cornified envelope involucrin and SPRR-2 protein expression in skin forms a protective cornified envelope structure at the terminal stage of keratocyte differentiation [17]. In the ocular surface, these proteins are upregulated when the epithelium is exposed to external injury resulting from desiccative stress [18]. This corneal and conjunctival epithelial keratinization response can severely impair visual function [19]. Furthermore, these proteins can disrupt tear film stability by entrapping soluble mucins produced by goblet cells [20]. We showed here that trehalose instillation prevented involucrin and SPRR-2 upregulation, which could account for increases in tear film stability.

Inflammation is a hallmark of DED since there are improved clinical signs in patients receiving topical corticosteroids [21] and cyclosporine A [22]. The increases in the Th1 [23] and Th17 [24-26] pathways appear to play a prominent role during chronic inflammation and tissue destruction in DED. Even though trehalose treatment does not change the balance between the T cell effectors and their regulators (Treg cells), the ability of the latter to suppress T cell activation seems to be affected in DED, especially for Th17. Th17 cells produce IL-17, which induces resident cell secretion of proinflammatory cytokines, such as IL-1, TNF-α, and IL-6 [27], and metalloproteinases such as MMP-9 [26] giving rise to corneal epithelial barrier disruption. These events are simulated in our model as were those of others since IL-1, TNF-α, and IL-6 were upregulated [28]. On the other hand, trehalose reduced proinflammatory cytokine upregulation compared to the PBS group. This decrease is noteworthy since PBS by itself is commonly used as a tear supplement in DED. This difference suggests that trehalose could offer a protective effect against the initiation and/or progression of inflammation in DED in addition simply providing lubrication and ocular surface hydration. Moreover, in the artificial tears field, very few compounds have been shown to present biologic anti-inflammatory functions. Except trehalose [8], only hyaluronan, in addition to his primary moisturizing properties, have been reported in in vitro studies or in clinical trials to present some anti-inflammatory efficiency by reducing inflammatory cytokines such as IL-6 and Il-8 [29] or an inflammatory-related antigen such as major histocompatibility complex class II human leukocyte antigen DR [30]. To our knowledge, no study has compared the anti-inflammatory biologic functions of these compounds.

Another indicator of DED is metalloproteinase gelatinase MMP-9 tear upregulation in a murine DED model [31] and in patients with DED [32]. Expression is low in healthy eyes [32] and is related to DED since the loss [33] and inhibition [34] of MMP-9 expression in DED models alleviate ocular surface disruption. MMP-9 cleaves to the epithelial basement membrane and tight junction proteins such as occludin [15], which maintain epithelial barrier function [16]. Upregulation of MMP-9 causes ocular surface epithelial cell detachment as well as tight junction disruption. Such changes may lead to increases in corneal barrier permeability. Trehalose treatment in the current study lowered corneal epithelial and conjunctival *MMP-9* gene expression more than that in mice that received no treatment or PBS supplementation instead. These considerations suggest that trehalose may lower *MMP-9* expression as a consequence of suppressing innate immune responses associated with increases in IL-1, TNF-α [35], and IL-17 [26] proinflammatory cytokine release, which induce *MMP-9* upregulation in DED.

Heat shock proteins such as HSP70 are upregulated by elevated temperature [36], hypoxia [37], oxidative stress [38] and desiccation [39]. HSP70 restores homeostasis by decreasing protein turnover and preserving conformation and function [40]. This adaptive response by HSP70 expression levels makes it a useful biomarker for assessing cell damage. We found that HSP70 expression in mice treated with trehalose was lower than that in the PBS and ICES groups, further indicating that trehalose lessens declines in ocular surface integrity resulting from exposure to the stresses imposed by the ICES.

In addition, it is widely accepted that trehalose can protect cells against desiccation. Trehalose was reported to be more efficient for treating dry eye syndrome than commercial eyedrops containing hyaluronan or hydroxyethylcellulose [41]. This beneficial effect is also attributable to trehalose’s ability to protect cell membranes [42] and proteins from oxidative injury by acting as a free-radical scavenger [43]. Furthermore, trehalose suppresses proinflammatory phenotype activation in macrophages in experimental septic shock [44] and in a model of peritoneal inflammation by protecting against I-kappa B-alpha dephosphorylation [45]. Trehalose supplementation also protects against apoptosis [9].

Taken together, the findings of the present study confirm that trehalose supplementation may provide a therapeutic option for treating ocular surface diseases such as DED.
